# Pyridoxal 5′-Phosphate Is a Slow Tight Binding Inhibitor of *E. coli* Pyridoxal Kinase

**DOI:** 10.1371/journal.pone.0041680

**Published:** 2012-07-25

**Authors:** Mohini S. Ghatge, Roberto Contestabile, Martino L. di Salvo, Jigar V. Desai, Amit K. Gandhi, Christina M. Camara, Rita Florio, Isabel N. González, Alessia Parroni, Verne Schirch, Martin K. Safo

**Affiliations:** 1 Department of Medicinal Chemistry, Institute for Structural Biology and Drug Discovery, Virginia Commonwealth University, Richmond, Virginia, United States of America; 2 Istituto Pasteur-Fondazione Cenci Bolognetti and Dipartimento di Scienze Biochimiche, Sapienza Università di Roma, Roma, Italy; 3 Consiglio Nazionale delle Ricerche, Istituto di Biologia Agroambientale e Forestale, Monterotondo Scalo, Roma, Italy; 4 Institute of Biocomputation and Physics of Complex Systems, Universidad de Zaragoza, Zaragoza, Spain; University of Cantebury, New Zealand

## Abstract

Pyridoxal 5′-phosphate (PLP) is a cofactor for dozens of B_6_ requiring enzymes. PLP reacts with apo-B_6_ enzymes by forming an aldimine linkage with the ε-amino group of an active site lysine residue, thus yielding the catalytically active holo-B_6_ enzyme. During protein turnover, the PLP is salvaged by first converting it to pyridoxal by a phosphatase and then back to PLP by pyridoxal kinase. Nonetheless, PLP poses a potential toxicity problem for the cell since its reactive 4′-aldehyde moiety forms covalent adducts with other compounds and non-B_6_ proteins containing thiol or amino groups. The regulation of PLP homeostasis in the cell is thus an important, yet unresolved issue. In this report, using site-directed mutagenesis, kinetic, spectroscopic and chromatographic studies we show that pyridoxal kinase from *E. coli* forms a complex with the product PLP to form an inactive enzyme complex. Evidence is presented that, in the inhibited complex, PLP has formed an aldimine bond with an active site lysine residue during catalytic turnover. The rate of dissociation of PLP from the complex is very slow, being only partially released after a 2-hour incubation with PLP phosphatase. Interestingly, the inactive pyridoxal kinase•PLP complex can be partially reactivated by transferring the tightly bound PLP to an apo-B_6_ enzyme. These results open new perspectives on the mechanism of regulation and role of pyridoxal kinase in the *Escherichia coli* cell.

## Introduction

Pyridoxal 5′-phosphate (PLP) is a cofactor for dozens of enzymes in the *E. coli* cell that are important in amino acid metabolism, as well as in several other pathways [1]. A *de novo* pathway for PLP biosynthesis occurs in *E. coli,* but a more important salvage pathway operates to recycle PLP during protein turnover [2]. The regulation of PLP homeostasis in *E. coli* and how each of the dozens of PLP requiring apo-B_6_ enzymes competes for available PLP, to form the catalytically active holo-B_6_ enzymes, are important unresolved problems.

**Figure 1:**
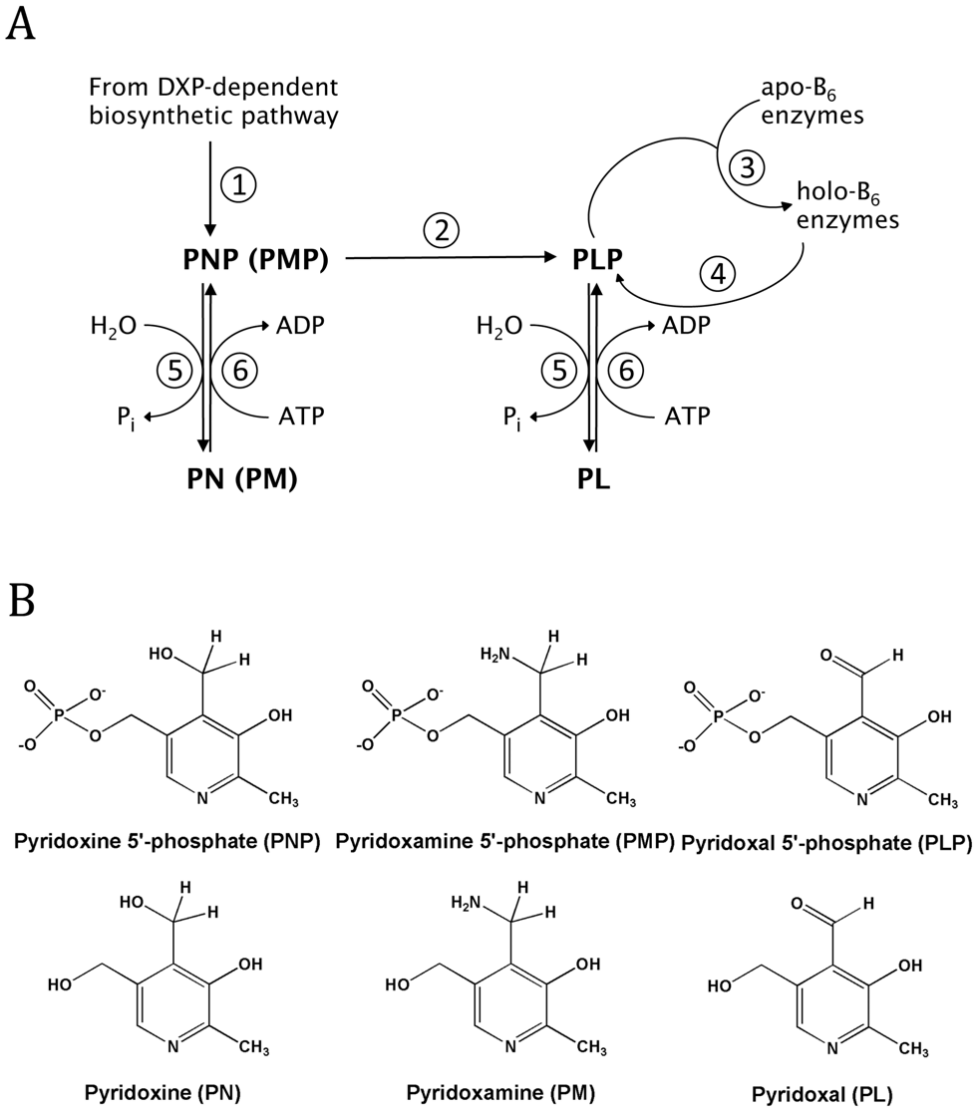
Vitamin B_6_ metabolism. A) Reactions in B_6_ metabolism. *Reaction 1*, enzymes involved in the *de novo* biosynthesis of pyridoxine 5′-phosphate (PNP); *Reaction 2*, PNP oxidase; *Reaction 3*, reaction of apo-B_6_ enzymes with PLP to form active holo-B_6_ enzymes; *Reaction 4*, degradation of holo-B_6_ enzymes to amino acids and PLP; *Reaction 5*, PLP phosphatase; *Reaction 6*, PL kinase. B) Structures of B_6_ vitamers.

Key reactions for maintaining PLP levels in the cell are shown in Figure 1. The biosynthetic pathway results in the formation of pyridoxine 5′-phosphate (PNP, reaction 1), which is converted to PLP by the flavin enzyme PNP oxidase (reaction 2) [3]. PLP adds to newly synthesized apo-B_6_ enzymes converting them to the catalytically active holo enzymes (reaction 3). PLP is released during protein turnover of B_6_ enzymes (reaction 4) and is converted to pyridoxal (PL) by cellular phosphatases (reaction 5) [3]–[5]. The formed PL is phosphorylated back to PLP by pyridoxal kinase (PL kinase) (reaction 6) [3]. Pyridoxine and pyridoxamine are also substrates for PL kinase (reaction 6). PL kinase, PNP oxidase and PLP phosphatase constitute the salvage pathway.

PLP is a reactive compound because the 4′-aldehyde forms aldimines with α-amines of amino acids and other compounds containing amino groups, the ε-amino group of lysine residues on non-B_6_ proteins, and thiazolidine adducts with sulfhydryl groups like cysteine. PL, which also contains the 4′-aldehyde moiety is much less reactive than PLP, since its aldehyde group, in aqueous solution and at neutral pH, exists mostly in the hydrated form [6], [7]. This reactivity of PLP poses two problems for the cell. First, to keep the cellular level of free PLP low so that it does not react with other nucleophiles, especially non B_6_-enzymes, and second, to supply enough PLP for the dozens of newly synthesized apo-B_6_ enzymes to form catalytically active holo-B_6_ enzymes. Some have proposed that the level of PLP is regulated and kept low by being an effective feedback inhibitor of both PL kinase [8] and PNP oxidase [9]–[15]. We report here on properties of *E. coli* PL kinase showing that PLP serves as a slow tight binding inhibitor of the enzyme.

The structure of PL kinase has been determined from several sources [11], [16]–[19]. In *E. coli* there are two PL kinases referred to as PL kinase1 and PL kinase2 [13], [20]–[22]. The activity of PL kinase2 is very low and there is a question if its function in the cell is to convert PL to PLP or if it is the enzyme for another unknown reaction. We have determined the structure and properties of both *E. coli* enzymes [11], [13]. This study reports on the properties of only *E. coli* PL kinase1 and we refer to it as *e*PL kinase. During our kinetic measurements, we observed that the enzyme rapidly loses activity as it catalyzes the conversion of PL and ATP into PLP and ADP. The reason for this inhibition is the subject of this report.

## Results

### Inactivation of *e*PL Kinase during Catalytic Turnover

**Figure 2:**
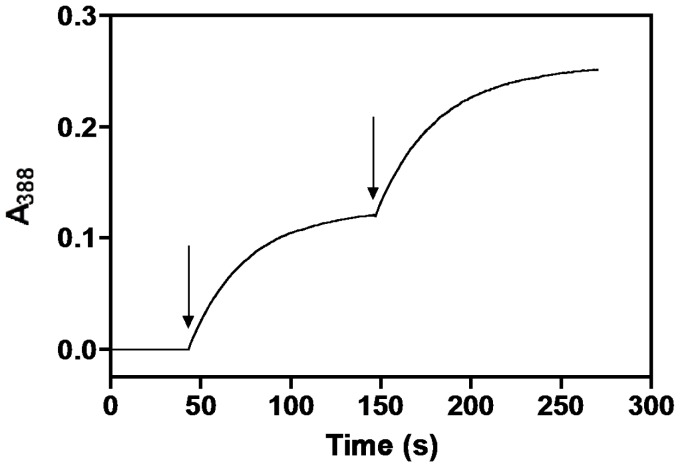
Kinetics of ePL kinase inhibition. The formation of PLP by ePL kinase was followed at 388 nm in 400 µl of reaction solution containing 1 mM MgATP, 0.2 mM MgCl_2_, and 1 mM PL at 37°C. At the first arrow, *e*PL kinase was added to 0.9 µM and PLP formation followed for about 120 seconds. A second aliquot of *e*PL kinase was added at the second arrow.

During our assay of *e*PL kinase to determine kinetic constants we observed a rapid loss of activity making kinetic studies from initial rates difficult. Figure 2 shows a typical assay for production of PLP. The rate of PLP formation is determined from the absorbance change at 388 nm. At pH 7.5, used in the assay, both PL and PLP have an absorption maximum at 388 nm, however, the extinction coefficient of PLP is much higher. From standard solutions, we calculated 5020 M^−1^ cm^−1^ to be the difference between the 388 nm molar extinction coefficients of the produced PLP and the consumed PL. The assay solution contained 1 mM MgATP, 1 mM PL and 0.9 µM *e*PL kinase. The rate of PLP formation decreased exponentially and after about 2 min the activity was near zero, with only 22 µM PLP being formed. The apparent rate of activity loss was around 1.5 min^−1^. Since a large excess of MgATP and PL was present in the assay, and the reaction is essentially irreversible, the termination of activity should not be the result of exhaustion of either substrate. However, it could be the result of product inhibition by ADP and PLP. To test if this was the reason for loss of activity, we added a second and equal amount of *e*PL kinase to the cuvette. The increase in absorbance at 388 nm resumed at the same rate as observed with the first addition of *e*PL kinase, showing that product inhibition was not the reason for the loss of activity, since at this point PLP is present in the reaction mixture. Furthermore, this second measurable activity also exponentially decreased at a rate of 1.5 min^−1^, so that after about 2 min there was no further increase in PLP production (Fig. 2). A third addition resulted in the same behavior (data not shown). The total absorbance change at 388 nm was the same for each addition of *e*PL kinase, showing that the amount of PLP produced was the same. We calculated the amount of PLP formed before the enzyme lost activity to be 22 µM, determined by dividing the absorbance change at 388 nm by 5020 M^−1^ cm^−1^. Since we also knew the concentration of the enzyme we could determine that about 25 catalytic turnovers were made before the enzyme was inactivated.

This experiment suggests that the loss of activity is the result of a partition between product release and an inactivation reaction.

### Properties of Inactive *e*PL Kinase

**Figure 3:**
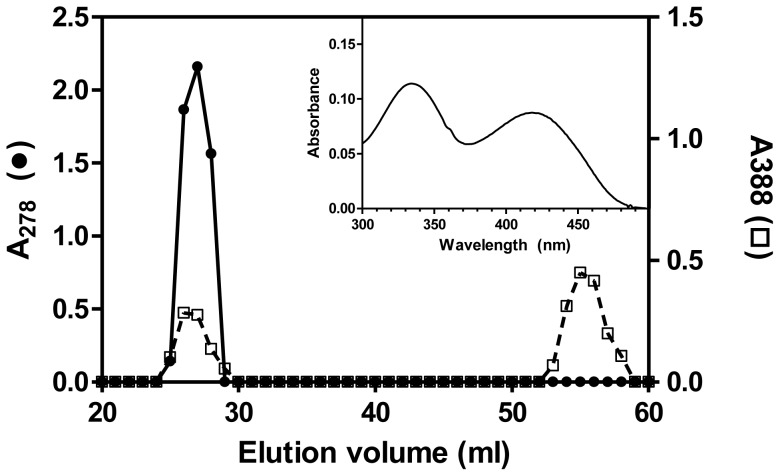
Formation of an ePL kinase•PLP complex. ePL kinase (300 µM) was incubated with MgATP (1 mM), PL (400 µM) and MgCl_2_ (0.2 mM) for 1 hour at 37°C in a volume of 2 ml. Then it was added to a 0.6 mm×45 cm column of Sephadex G-50 equilibrated with 1 mM MgATP in reaction buffer and eluted with equilibration solution. Aliquots of 400 µl were collected and absorbance at 278 nm (*e*PL kinase) and 388 nm (PLP) was recorded. Inset: spectrum of *e*PL kinase with bound PLP showing that the bound PLP exhibits absorbance peaks at 336 nm and 420 nm.

In order to check if the observed inhibition was reversible, we incubated *e*PL kinase (300 µM) with PL (400 µM) and MgATP (1 mM) for one hour at 37°C. The protein was then separated from excess substrates and products by size exclusion chromatography on a Sephadex G-50 column. As shown in Figure 3, the fractions containing the protein showed an absorbance at 388 nm (beside the 280 nm absorption peak), suggesting that PLP was bound to *e*PL kinase. Addition of NaOH to a final concentration of 0.2 M to the protein samples resulted in a spectrum identical to the spectrum of PLP in alkaline solution (data not shown). From the observed absorbance maxima at 388 nm and 278 nm for PLP and protein, respectively, we determined that the stoichiometry of PLP to *e*PL kinase subunits was 1∶1.

What is of interest in the elution profile shown in Figure 3 is the lack of a tailing edge in the 388 nm absorption profile of the chromophore that co-elutes with the protein in the size-exclusion chromatography. This suggests that the complex between protein and PLP is very tight and does not readily dissociate upon the slow migration of the *e*PL kinase down the sizing column. The spectrum of a typical fraction containing protein-bound PLP is shown in the inset of Figure 3 and exhibits two distinct absorbance maxima at 336 nm and 420 nm. The band with maximum at 420 nm is characteristic of PLP bound as an aldimine [23]. The aldimine linkage (or Schiff base) between PLP and a lysine residue is a reversible covalent bond. It is characteristic of PLP bound to an ε-amino group of an active site lysine residue in holo-B_6_ enzymes [24].

### Rate of Formation of *e*PL Kinase•PLP Complex

**Figure 4:**
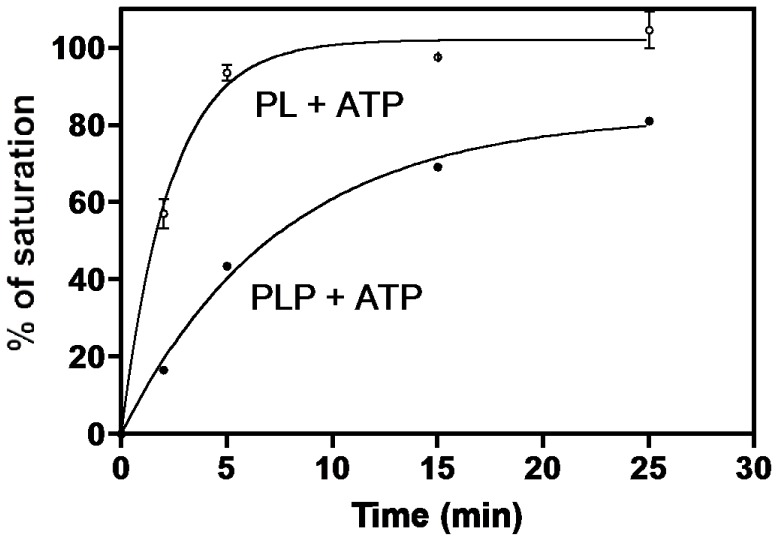
Rate of formation of ePL kinase•PLP complex. To a series of 100 µl solutions in Eppendorf vials containing 0.4 mM MgATP, 0.20 mM MgCl_2_ and either 0.150 mM PL or 0.150 mM PLP at 37°C was added 9 nmoles of *e*PL kinase (90 µM). After 2, 5, 15 and 25 min contents of vials were withdrawn and placed on small Sephadex G-50 columns at 4°C equilibrated with 1 mM MgATP and 0.2 mM MgCl_2_ (see Experimental Procedures) to separate bound and free PLP. The eluate of each column was 400 µl. Spectra were recorded and the absorbance at 420 nm determined. Open circles, reactions initiated with PL, closed circles reactions initiated with PLP. The lines through the experimental points are those obtained from nonlinear least squares fittings of data to an exponential equation which gave rate constants of 0.4 min^−1^ and 0.1 min^−1^ and amplitudes of 100% and 83% for the experiments initiated with PL and PLP, respectively.

The considerable separation between the *e*PL kinase•PLP complex and free PLP, shown in Figure 3, provides a method to rapidly separate the enzyme from unbound substrates and products by using small spin columns (see Experimental Procedure). To determine the rate at which the complex was formed, *e*PL kinase (90 µM) was incubated with 400 µM MgATP and 150 µM PL at 37°C from 2 to 25 min. At several time intervals, a sample of the reaction mixture was removed and added to a small spin Sephadex G-50 column, kept at 4°C, to rapidly separate enzyme-bound PLP from unbound small molecules. The absorbance of the eluted enzyme at 420 nm and 336 nm was measured to determine the amount of bound PLP and the percentage of saturation per enzyme subunit. As shown in Figure 4 (open circle) the amount of bound PLP reached about 100% of saturation in about 10 minutes, with 50% saturation occurring in less than 2 min. However, if *e*PL kinase was incubated with 150 µM PLP, in place of PL, and 400 µM MgATP the amount of bound PLP after 10 minutes was about 60% and reached only 80% of saturation in 25 min. In this case, it took about 7.5 min to reach 50% saturation (Fig. 4, solid circle). This shows that PLP generated by enzyme turnovers leads to faster formation of the complex compared to when PLP is added as an external ligand.

The *e*PL kinase concentration in the previous experiment with PL and MgATP is high and the amount of time required to process the reaction would convert much of the PL to PLP and an equivalent amount of the MgATP to MgADP, suggesting that the complex may contain both PLP and MgADP. The experiment was repeated with PLP and MgADP, and the rate of formation of the complex was similar to the rate observed with PL and MgATP (Table 1). On the other hand, when PLP and Mg^2+^ were used in the absence of any nucleotide the rate was slower and comparable to the rate observed with PLP and MgATP, shown in Figure 4 (Table 1). This suggests that a tight complex is formed with PLP and MgADP. The spectrum of the *e*PL kinase•PLP complexes in these experiments was the same as shown in the inset of Figure 3.

**Table 1:** Rate of formation of *e*PL kinase•PLP complex in the presence of different substrates and products^a^.

Substrates and products added to *e*PL kinase	Rate of complex formation (min^−1^)
PL + MgATP	0.43
PLP + MgATP	0.13
PLP + MgADP	0.31
PLP	0.16

a *e*PL kinase (90 µM) samples were incubated with different combinations of PL (150 µM), PLP (150 µM), MgATP (400 µM), MgADP (400 µM) and MgCl_2_ (0.2 mM). Complex formation was measured at time intervals as described in Materials and Methods section. Values are the average of three independent measurements and the standard deviation was less than 10%.

Keeping in mind that *e*PL kinase also phosphorylates PN and PM, we repeated the same experiments described above, to determine if PNP and PMP also formed a tight complex with *e*PL kinase. Our results showed that a 25 min incubation at 37°C resulted in no detectable complex formation with these two vitamers (data not shown).

### Nucleotide Ligands in the *e*PL Kinase•PLP Complex

The experiments described in the previous section had either MgATP or MgADP present at all times during the chromatographic separation of free PLP from the kinase•PLP complex. To determine how important MgATP, MgADP and MgCl_2_ were in forming the complex, and whether the nucleotides were also bound in the complex, we performed the incubation of *e*PL kinase with several different combinations of these compounds, and carried out the final separation of the enzyme from the small molecules in the absence of purine nucleotides. After the separation, we analyzed the complexes for the presence of PLP, MgATP and MgADP, after complete denaturation by 0.2 M NaOH (see Experimental Procedures). The results are reported in Table 2. PLP was always found to be present in the complex, while ATP was not detected in any of the analyzed complexes. On the other hand, ADP was present in the complexes, but not in a stoichiometric concentration with respect to enzyme subunits. Apparently, ADP is not bound as tightly as PLP to *e*PL kinase. PLP, in the absence of nucleotides or MgCl_2_, binds tightly to *e*PL kinase, although its binding is further promoted by the inclusion of exogenous MgCl_2_, suggesting that Mg^2+^ plays a role in forming the tight complex (Table 2). These results show that the tight complex in the presence of nucleotides is actually *e*PL kinase•PLP•MgADP. However, in the following experiments we focused our attention on the PLP moiety of the complex and therefore will refer to the *e*PL kinase•PLP complex.

**Table 2:** Measurement of nucleotides in the *e*PL kinase•PLP complexes^a^.

			%PLP	%ATP	%ADP
PL	MgATP	MgCl_2_	95	n.d.^b^	21
PLP	MgATP	MgCl_2_	100	n.d.	n.d.
PLP	MgADP	MgCl_2_	72	n.d.	43
PLP	–	MgCl_2_	82	–	–
PLP	–	–c	51	–	–

a *e*PL kinase (130 µM) samples were incubated with different combinations of PL (0.5 mM), PLP (0.5 mM), MgATP (1 mM), MgADP (1 mM) and MgCl_2_ (0.5 mM) for one hour at 37°C. These samples were then passed through a sizing column and the fractions containing the enzyme were collected and analyzed with respect to PLP and nucleotides content. The values reported in the table are percentages of saturation with respect to enzyme subunits.

b n.d.: not detectable with the method employed (minimum detectable level 1.5 µM; see Experimental Procedures for details).

c MgCl_2_ was also omitted from the chromatography equilibration and elution buffer.

### Rate of Dissociation of the *e*PL Kinase•PLP Complex

**Figure 5:**
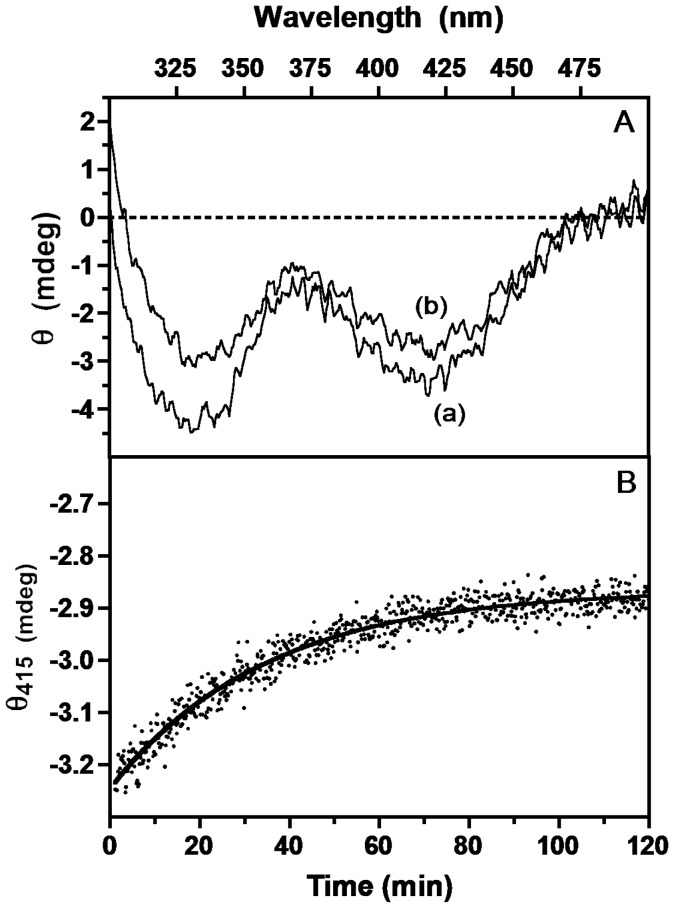
Rate of dissociation of PLP from the ePL kinase•PLP complex. The rate of dissociation of PLP from the *e*PL kinase•PLP complex was followed by observing the change in optical activity of the bound PLP at 37°C. Panel A: Spectra of the complex (60 µM) at time zero (curve a) and after 120 min in the presence of 10 µM PLP phosphatase (curve b). Panel B: measured decrease in optical activity after addition of PLP phosphatase at 415 nm with time and as an exponential process with rate constant of 0.012 min^−1^.

To determine the rate of dissociation of PLP from the *e*PL kinase•PLP complex, we took advantage of the property that free PLP does not elicit an optical signal in a CD spectrum because it is a symmetrical molecule. But, when PLP is bound to B_6_ enzymes, the asymmetric environment at the active site induces a CD signal in PLP that can be either negative or positive [25]. A *e*PL kinase•PLP complex was obtained by incubating 130 µM *e*PL kinase with 0.5 mM PL and 1 mM MgATP, and separated on a sizing column in the absence of purine nucleotides. The CD spectrum of the obtained *e*PL kinase•PLP complex exhibits negative ellipticity bands at about 330 nm and 415 nm (Fig. 5A, curve (a)). To determine the rate of PLP dissociation from the complex we followed the rate of decrease of the CD signal at 415 nm when the complex was incubated with a specific PLP phosphatase (10 µM) [26]. As PLP dissociates from the complex, the phosphatase rapidly converts it to PL and this results in a decrease of the CD signal. The observed kinetics (Fig. 5B) shows that the rate of dissociation is very slow (0.012 min^−1^) and fits very well to a first-order decay curve. The final CD spectrum after 120 min shows that only a fraction of the bound PLP was released (Fig. 5A, curve (b)). Measurement of the residual PLP in the sample showed that about 60% of the initially bound PLP was present even after a 2-hour experiment. Very similar results were obtained using alkaline phosphatase in place of PLP phosphatase. In another experiment, an identical *e*PL kinase•PLP complex was reduced with NaBH_4_. This potent reductant is often used to reduce the PLP aldimine double bond (absorbing at 420 nm) to a single bond. The reduction is accompanied by a large spectral change. Also in this case, the reduction of only a small fraction of the enzyme-bound PLP was observed (as a decrease of the 420 nm absorbing band and a concomitant appearance of a band at around 325 nm). Evidently, there is a fraction of tightly bound PLP that does not dissociate from *e*PL kinase and is not easily accessible from the solvent by NaBH_4_.

### Mechanism of Formation of *e*PL Kinase•PLP Complex

**Figure 6:**
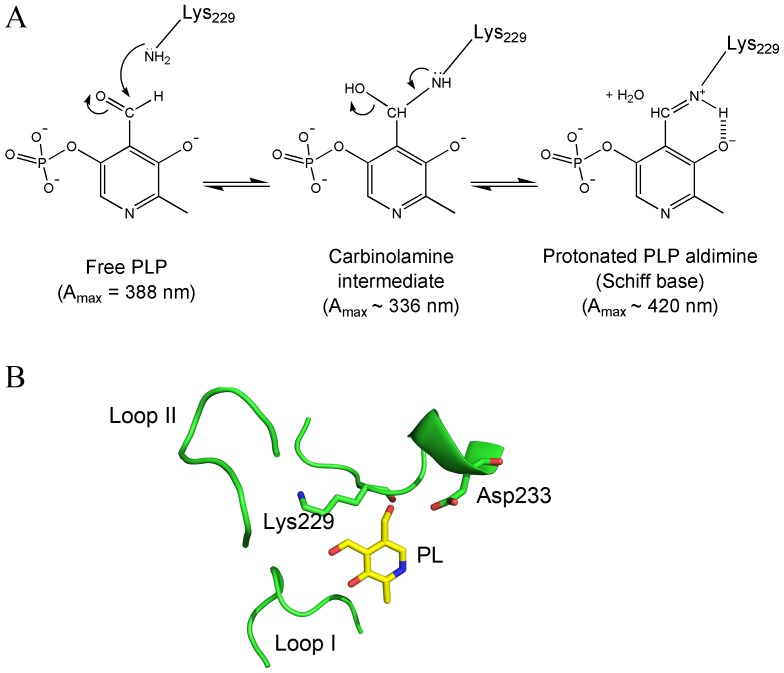
Mechanism of reaction between PLP and the active site K229. A) A scheme showing the structures of the carbinolamine intermediate and the enolimine form of the protonated PLP aldimine. B) Active site structure of the binary complex of *e*PL kinase and PL showing the position of K229.

The spectrum of the *e*PL kinase•PLP complex shown in Figure 3 shows absorption peaks at 336 nm and 420 nm. These absorbance maxima are also represented in the CD spectra as observed in Figure 5A. The peak at 420 nm is characteristic of a protonated PLP aldimine and therefore clearly indicates that PLP is covalently bound, albeit reversibly to a lysine residue of the enzyme [23]. The absorbance of the peak at 336 nm could also be attributed to a covalently bound PLP, most likely the carbinolamine intermediate which occurs in the aldimine linkage formation (Fig. 6A). One has to keep in mind that an aldimine linkage is a readily reversible covalent bond and therefore PLP is still able to dissociate from the enzyme. The structure of *e*PL kinase shows the presence of a lysine residue, namely Lys229 (K229), near the active site. In the published crystal structure of *e*PL kinase with bound PL, the side chain of K229 is about 4.5 Å from PL, but its side chain can rotate to place the ε-amino group ∼1.6 Å from the aldehyde group of PL. (Fig. 6B) [11]. This suggests a possible conformational change of the lysine side-chain position to form the Schiff-base linkage with the PLP. We have not been able to obtain crystals of the *e*PL kinase•PLP complex to ascertain if PLP is actually bound to this lysine residue. Therefore, we decided to change this residue to a glutamine to determine if K229 was critical for catalytic activity, enzyme inactivation, tight binding of PLP and the spectral properties shown in Figures 3 and 5A.

**Figure 7:**
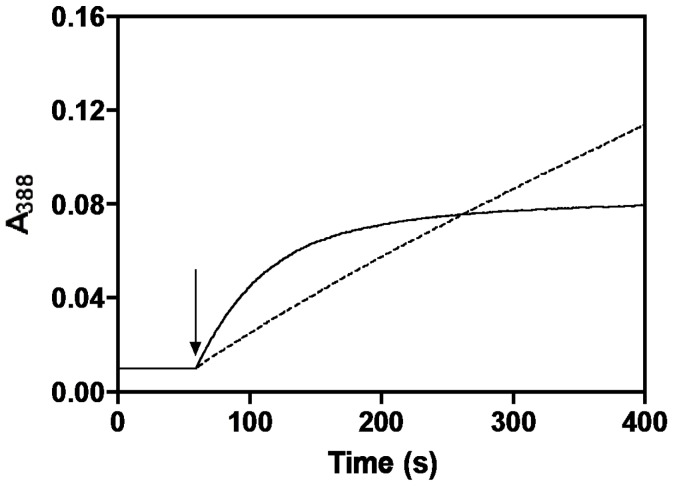
Comparison of PLP formation with wild type and K229Q ePL kinases. The kinetics of PLP formation catalyzed by *e*PL kinase was followed at 388 nm upon addition (shown by the arrow) of 0.3 µM of wild type enzyme (continuous line) or 0.3 µM of K229Q enzyme (dotted line). Each reaction contained 1 mM MgATP, 0.2 mM MgCl_2_ and 1 mM PL, at 37°C.

Previous studies may have given erroneous kinetic constants for *e*PL kinase because they were not appreciative of the rapid loss of activity due to PLP inhibition, as shown in Figure 2. We have more carefully determined the kinetic constants for the wild type enzyme by determining the initial rate in the first few seconds before inhibition becomes a factor. The results are shown in Table 3. The K229Q mutant enzyme was expressed and purified as described for the wild type enzyme. Kinetic constants were determined and are recorded in Table 3. These results show that K229 plays a role in both binding of substrates and catalysis but is not essential for activity. Note that the affinity for MgATP in K229Q enzyme is greater compared to wild type *e*PL kinase. When the mutant enzyme is incubated with PL and MgATP and passed down a sizing column, no PLP is found tightly bound as shown in Figure 3 for wild type *e*PL kinase. Furthermore, unlike wild type *e*PL kinase, incubation of K229Q with PL and MgATP does not result in a rapid loss of activity (Fig. 7).

**Table 3:** Kinetic parameters for wild type and K229Q mutant *e*PL kinase.

	K_m_ for PL (µM)	K_m_ for MgATP (µM)	k_cat_ (min^−1^)
wild type	60	460	240
K229Q	384	122	26

### Reactivation of Inhibited *e*PL Kinase•PLP Complex

If the inactivation of *e*PL kinase takes place in an *E. coli* cell, how does the kinase continue to function in order to generate PLP? Is there a mechanism by which the tightly bound PLP is removed to reactivate the enzyme? Previous studies with PNP oxidase and PLP synthase have been shown to bind PLP tightly. For both enzymes this tightly bound PLP was transferred to an apo-B_6_ enzyme, apo serine hydroxymethyltransferase (apo-SHMT) for PNP oxidase and aspartate aminotransferase for PLP synthase [27], [28]. To test if an apo-B6 enzyme could also regenerate an active *e*PL kinase we added purified apo-*E. coli* SHMT (*e*SHMT) and determined if the tightly bound PLP on the kinase was transferred to the apo-*e*SHMT to form the active holo-*e*SHMT. *e*SHMT is a good B_6_ enzyme to use since the active enzyme forms an abortive complex with product glycine and substrate tetrahydrofolate that exhibits an absorption peak at 495 nm with a large molar absorbance coefficient [27]. The rate of formation of this absorbing complex can be followed continuously with time.

**Figure 8:**
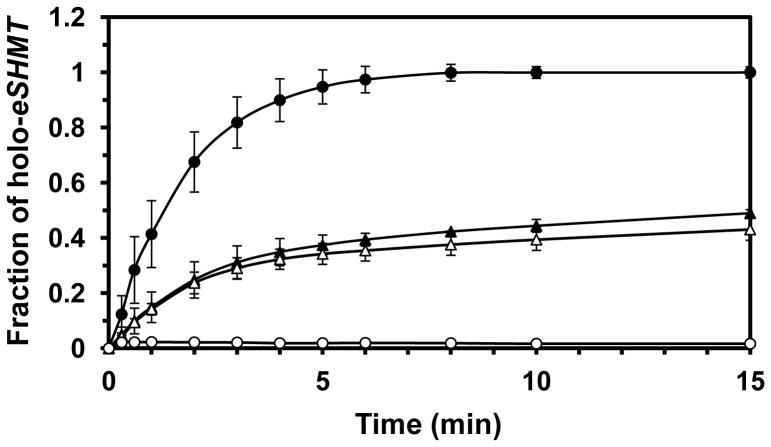
Rate of transfer of PLP from PL kinase•PLP to apo E. coli serine hydroxymethyltransferase. Fraction of apo-*e*SHMT (20 µM) being converted to holo-*e*SHMT with PLP (20 µM) (•–•). Fraction of apo-*e*SHMT (20 µM) being converted to holo-*e*SHMT with an equivalent amount of PL kinase•PLP (20 µM) (▴–▴). Repeat of the conversion of apo-*e*SHMT with free PLP (○–○) or PL kinase•PLP (Δ–Δ) to holo-*e*SHMT in the presence of 3 µM PLP phosphatase.

As shown in Figure 8, when an equivalent subunit concentration of apo-*e*SHMT is added to *e*PL kinase•PLP complex, there is a transfer of PLP to form holo-*e*SHMT. The rate of transfer far exceeds the rate of dissociation of PLP from the complex as determined by CD studies (Fig. 5). Of interest is the observation that only 50% of the tightly bound PLP is transferred, as suggested by the effect of the addition of an excess of free PLP to apo-*e*SHMT. The addition of a phosphatase did not reduce the amount of transfer of PLP suggesting PLP was being transferred directly without coming into contact with the phosphatases. Although speculative, it is possible the PLP is not even released into the solvent but directly channeled to apo-*e*SHMT. We observed that some catalytic activity of PL kinase returns upon transfer of the tightly bound PLP to apo-*e*SHMT but is much less than the expected 50% (data not shown). Future studies are focused on understanding in much more detail the mechanism of transfer of PLP and the reactivation of PL kinase.

## Discussion

There are three known enzymes in living systems that catalyze the production of PLP: PNP oxidase [3], [29], present in both prokaryotic and eukaryotic organisms; PL kinase, which is also widely distributed in nature [3], [11]; and PLP synthase, which is found in plants and many microorganisms [30]. Both PNP oxidase and PLP synthase have been shown to bind PLP tightly and to transfer the tightly bound PLP to an apo-B_6_ enzyme [28], [31]. This report is the first study on the properties of the formation and dissociation of a tightly bound PLP in *e*PL kinase.

Three classes of enzyme competitive inhibitors have been described [32]: those which inhibit rapidly, those which inhibit rapidly followed by a slow conformational change, and those which inhibit slowly. Classical competitive inhibitors act rapidly and show a high affinity for the active site of the ground state enzyme, whereas slow binding inhibitors show a high affinity for an intermediate state of the enzyme. Slow binding inhibition is characterized by an initial weak binding to the ground state enzyme, followed by tighter binding to the transition state structure. In general, this type of inhibition is considered more physiologically relevant since upstream accumulation of the substrate cannot relieve the inhibition brought about by this form of inhibition [33].

The results presented here suggest that PLP is a slow tight binding inhibitor of *e*PL kinase. The mechanism of inhibition consists in the formation of a Schiff base between PLP and an active site lysine residue (Fig. 6A). The inactivation of the enzyme is faster when both PLP and MgADP are present, compared to when PLP is present alone or together with MgATP (Fig. 4 and Table 1). Therefore, the inhibition occurs more rapidly during the catalytic turnover of the enzyme, in which the enzyme may go through an intermediate state whose conformation favors the covalent binding of PLP. It appears that during the catalytic cycle, or when both PLP and MgADP are bound, the active site of *e*PL kinase is in a conformation that places the ε-amino group of K229 in a favorable position to form a covalent bond with C4′ of PLP. The position of K229 in the active site structure of the unliganded *e*PL kinase is shown in Fig. 6B. Formation of an aldimine between PLP and the ε-amino moiety of K229 is suggested by the absorption maximum at 420 nm (Fig. 3 inset) and by the failure of PLP to bind tightly to K229Q *e*PL kinase and to inhibit its activity (Fig. 7). The 336 nm absorbing band of the tightly bound PLP (Fig. 3, inset) can be accounted for by several possible structures. One of the most probable is a carbinolamine intermediate, which occurs during the formation of the aldimine (Fig. 6A). In the carbinolamine structure, the C4′ carbon of PLP is tetrahedral because of the addition of the ε-amino moiety of K229 across the double bond to oxygen. Another possible structure is the enolimine tautomer of the PLP protonated aldimine also found at the active site of PLP-dependent enzymes [25], [34].

The rate of dissociation of PLP from the *e*PL kinase•PLP complex is very slow, as shown by the CD studies in the presence of specific and non-specific PLP phosphatases (Fig. 5A and B). This slow rate cannot account for the order of magnitude faster rate of transfer of the tightly bound PLP to apo-*e*SHMT.

Our results raise questions about the role of *e*PL kinase *in vivo*. The observed inhibition mechanism and the transfer of PLP to apo-B_6_ enzymes may be a strategy to tune *e*PL kinase activity on the actual requirements of the PLP cofactor. Moreover, since PLP is such a reactive compound, having it bound tightly to *e*PL kinase would afford protection against unwanted side reactions, in which it can be dephosphorylated or form aldimines with free amino acids or ε-amino groups on lysine residues in non-B_6_ proteins. We observed that the tightly bound PLP is protected from dephosphorylation by either a specific PLP phosphatase or alkaline phosphatase. But if protecting PLP from the unproductive side reactions is the purpose of its tight binding, then there must be a mechanism by which PLP is released to activate the newly synthesized apo-B_6_ enzymes, restoring the catalytic turnover of the kinase.

## Materials and Methods

### Materials

All buffers, reagents and chromatography materials were of the purest grade available and are the same as previously reported [11], [20]. *e*PL kinase was expressed, purified and stored as previously described [11]. Calf intestine alkaline phosphatase was purchased from Fermentas International Inc.

### Methods

All experiments were carried out in 20 mM potassium HEPES, pH 7.5, containing 0.2 mM MgCl_2_ except when otherwise stated. The catalytic activity of *e*PL kinase was determined spectophotometrically at 37°C by observing the initial increase in absorbance at 388 nm during the conversion of PL to PLP [20].

### Purification of PLP Phosphatase

Human brain PLP phosphatase cDNA cloned in pET19b was kindly provided by Dr. Anette Bøe, University of Bergen, Norway [35]. The plasmid was transformed into *Escherichia coli* strain Rosetta (λDE3) pLysS. The recombinant cells were grown at 37°C in LB broth with ampicillin (50 µg/ml) and chloramphenicol (34 µg/ml) until OD_600_ was about 1.0 and induced with 50 µM isopropyl-β-D-thiogalactopyranoside (IPTG). The cells were grown for additional 24 hours at 18°C and then harvested by centrifugation. The His-tagged enzyme was released by homogenization in 50 mM sodium phosphate buffer, pH 8, containing 300 mM NaCl and 10 mM imidazole. After centrifugation the cell extract was added to a 10 ml Ni-NTA agarose column (Qiagen) and the column was washed sequentially with 20, 30, 50, 75, 150 mM imidazole buffers, pH 8, until the absorbance at 280 nm was less than 0.1. The PLP phosphatase was then eluted with 250 mM imidazole buffer. The purified enzyme was dialyzed against 160 mM NaCl and 4 mM MgCl_2_ in 40 mM sodium BES, pH 7.0 for 4 hours. A second dialysis in the same buffer, except with 80 mM NaCl, was continued overnight. The enzyme was stored at −20°C. About 20 mg of 90% pure enzyme was obtained per liter of culture. Activity was determined by monitoring the decrease in absorbance at 388 nm during the conversion of PLP to PL [4].

### Preparation of K229Q *e*PL Kinase

In order to avoid any contamination from wild type *e*PL kinase from the host cells, the K229Q mutant was expressed as a His-tagged protein and purified by affinity chromatography. The addition of the His-tag at the C-terminus of *e*PL kinase was obtained by subcloning the coding sequence of wild type *e*PL kinase [20] into pET28a plasmid (Novagen), between *Nde*I and *Eco*RI restriction sites.

The K229Q mutant was prepared by site-directed mutagenesis using the pET28-*e*PL kinase construct as template, by the Quick-Change™ method from Stratagene. Two oligonucleotides (synthesized by MWG-Biotech) containing the mutations were used as primers: 5′-GGTTAAAACTGACCTGCAAGGGACTGGCGACC-3′ and its complementary oligonucleotide (the mutated bases are underlined). *E. coli* DH5α cells were transformed and used to amplify the mutated plasmid. Both strands of the coding region of the mutated gene were sequenced. The only differences with respect to wild type were those intended.

Enzyme expression of the His-tagged form of the enzyme, performed using the HMS174(λDE3) strain of *E. coli*, was induced by adding 0.2 mM IPTG. After incubation at 28°C with constant agitation for 20 h, cells were harvested and suspended in 50 mM sodium phosphate buffer, pH 8, containing 0.3 M NaCl. Cells were ruptured by sonication. Following the addition of DNase I (0.02 mg/ml), the lysate was centrifuged, and the supernatant was loaded on a 25 ml Ni-NTA agarose column, equilibrated with lysis buffer. The column was washed with the same buffer, containing 40 mM imidazole, and the enzyme was then eluted with a 400-ml linear gradient of imidazole from 40 mM to 0.5 M. Fractions containing the enzyme were detected by SDS-polyacrylamide gel electrophoresis, pooled and dialyzed against 20 mM potassium HEPES, pH 7.5. The His-tagged form of the wild type enzyme was also purified and characterized with respect to its catalytic properties, which were found to be identical to the original form of the enzyme.

### Rate of Formation of *e*PL Kinase•PLP Complex in the Presence of Different Substrates and Products

The rates of formation of *e*PL kinase•PLP complex were determined by small Sephadex G-50 spin columns as follows. One ml plastic syringes with a glass wool plug were filled to the 0.5 ml mark with Sephadex G-50 in 50 mM potassium HEPES, pH 7.5. In an Eppendorf tube, 90 µM *e*PL kinase was incubated for various times with 0.4 mM MgATP or MgADP, 0.150 mM PL or PLP, 0.2 mM MgCl_2_, in 0.1 ml reaction volume at 37°C. After incubation, each sample was placed on the G-50 column that was equilibrated with buffer, which contained either 1 mM MgATP or 1 mM MgADP and 0.2 mM MgCl_2_ in 20 mM potassium HEPES buffer, pH 7.5, at 4°C. In the experiment in which only PLP was present, the nucleotides were omitted from the buffer. The sample was allowed to soak into the column. To each column, 400 µl of equilibration solution at 4°C was added and the plastic syringes (placed in a glass test tube) were inserted in a swinging bucket centrifuge and rotated at about 150 rpm. The buffer containing the protein elutes into the glass tube and the small molecules remain on the column. The 400 µl of eluted sample is then placed in a 1 cm cuvette and the spectrum recorded. The total time to separate *e*PL kinase from small molecules is about 3 min. Controls show that 89±3.5% of *e*PL kinase eluted in the 400 µl and less than 2% of the excess PL and PLP were eluted.

### Stoichiometry of *e*PL Kinase•PLP Complexes

The stoichiometry of the formation of the *e*PL kinase•PLP complex was determined by adding NaOH to a final concentration of 0.2 M to denature the protein and release the tightly bound PLP. The absorbance at 388 nm was used to determine the concentration of the cofactor, that in 0.2 M NaOH exhibits a molar extinction coefficient of 6600 M^−1^ cm^−1^ [7]. The concentration of *e*PL kinase in the complex was also determined in 0.2 M NaOH, using an extinction coefficient at 292 nm (a wavelength at which PLP has negligible absorbance) of 35,416 M^−1^ cm^−1^, which was determined from the absorption spectrum of a protein sample whose concentration was previously determined in buffer (extinction coefficient at 278 nm of 28,850 M^−1^ cm^−1^) [20]. The concentration of PLP in the buffer at pH 7.5 was calculated using an extinction coefficient at 388 nm of 5305 M^−1^ cm^−1^. This coefficient was derived from the absorption spectrum of a PLP sample whose concentration was determined in NaOH.

### Determination of ATP and ADP Concentration in *e*PL Kinase•PLP Complexes

The complexes were obtained by incubating 130 µM *e*PL kinase samples with different combinations of PLP, PL, MgATP, MgADP and MgCl_2_ as detailed in Table 2, at 37°C for 1 hour in 20 mM potassium HEPES, pH 7.5. The samples were then passed through a G-50 Sephadex column (1.3 cm×24 cm) that had been previously equilibrated with 20 mM potassium HEPES, pH 7.5, containing 0.5 mM MgCl_2_ and eluted with the same buffer. Fractions containing *e*PL kinase were pooled. PLP and protein concentrations were measured as described above. In order to determine nucleotide concentration, 600 µL of sample was treated as follows. The protein was denatured by the addition of 0.2 M NaOH in order to allow the release of protein-bound molecules. Addition of 0.2 M HCl neutralized the solution followed by centrifugation to remove the protein precipitate. The supernatant (450 µL) was transferred in a clean tube and assayed for the presence of ATP and ADP. ADP concentration was measured, by coupling pyruvate kinase and lactate dehydrogenase reactions, from the absorbance change at 340 nm due to NADH oxidation. A typical assay, carried out at 37°C in 20 mM potassium HEPES, pH 7.5, in a final volume of 600 µL, contained 450 µL of the complex sample, 2 mM phosphoenolpyruvate, 0.25 mM NADH, 18 units of D-lactate dehydrogenase and 10 units of pyruvate kinase. ATP concentration was determined using human PL kinase activity, measuring the absorbance change at 388 nm due to PLP formation from PL. Assays, carried out at 37°C in 20 mM potassium HEPES, pH 7.5, in a final volume of 600 µL, contained 450 µL of the complex sample, 0.17 mM PL and 8 µM PL kinase. Both assays had been previously validated using commercial nucleotide samples of known concentrations, which ranged from 1.5 µM to 40 µM, obtaining two calibration curves for ATP and ADP measurements. The minimum detectable nucleotide concentration was estimated to be around 1.5 µM for both assays.

### Rate of Dissociation of PLP from the *e*PL Kinase•PLP Complex

The CD spectrum of bound PLP in the ternary complex was used to determine the rate of dissociation of PLP. Bound PLP in the complex gives negative bands at about 336 nm and 415 nm, but free PLP does not exhibit a CD signal since it is a symmetrical molecule. A 60 µM solution of *e*PL kinase•PLP complex was placed in a 1 cm cell and the signal at 415 nm followed with time at a bandwidth of 5 nm at 37°C. Included in the sample was a 10 µM solution of PLP phosphatase or alkaline phosphatase to rapidly convert any released PLP to PL.

### Determination of PL Kinase Kinetic Constants for Wild Type and K229Q *e*PL Kinases

All assays were performed at 37°C in a 1 cm thermostated cuvet. The increase of absorbance at 388 nm, due to the difference between PLP production and PL consumption (ε = 5020 M^−1^ cm^−1^ at pH 7.5), was monitored for about 60 s in an Agilent 8453 spectrophotometer. The very first initial velocity was used to calculate kinetic parameters in order to avoid enzyme inhibition. K_m_ and k_cat_ values for ATP and PL were determined as previously described [20].

### Transfer of PLP from the *e*PL Kinase•PLP Complex

The complex was prepared as described in the section on the analysis of ADP and ATP. In a 1 ml cuvette in HEPES buffer, pH 7.5, 37°C, the following was added; glycine (50 µM), tetrahdyrofolate (100 µM); and apo-*e*SHMT (20 µM). The transfer was started by the addition of the 20 µM PL kinase*•*PLP. Transfer of PLP from the PL kinase complex was followed by the formation of the holo-SHMT•Gly•tetrahdyrofolate abortive complex that absorbs at 495 nm [27]. The first experiment was to determine if any of the PLP in the complex was transferred by following the absorbance at 495 nm. To determine the fraction of holo-*e*SHMT formed a control was performed where the PL kinase complex was replaced with 25 µM PLP, which would fully saturate the apo-*e*SHMT giving the A495_nm_ for fully saturated holo-*e*SHMT. Each of these two experiments was duplicated in the presence of 3 µM PLP phosphatase that was added to the reaction solution prior to starting the transfer with either free PLP or PL kinase*•*PLP.

## References

[1] Eliot AC, Kirsch JF (2004) Pyridoxal phosphate enzymes: Mechanistic, structural, and evolutionary considerations. Annu Rev Biochem 73: 383–415. 15189147 10.1146/annurev.biochem.73.011303.074021

[2] di Salvo ML, Contestabile R, Safo MK (2011) Vitamin B(6) salvage enzymes: Mechanism, structure and regulation. Biochim Biophys Acta 1814: 1597–1608. 21182989 10.1016/j.bbapap.2010.12.006

[3] McCormick DB, Chen H (1999) Update on interconversions of vitamin B-6 with its coenzyme. J Nutr 129: 325–327. 10024608 10.1093/jn/129.2.325

[4] Jang YM, Kim DW, Kang TC, Won MH, Baek NI, et al (2003) Human pyridoxal phosphatase. molecular cloning, functional expression, and tissue distribution. J Biol Chem 278: 50040–50046. 14522954 10.1074/jbc.M309619200

[5] Clayton PT (2006) B6-responsive disorders: A model of vitamin dependency. J Inherit Metab Dis 29: 317–326. 16763894 10.1007/s10545-005-0243-2

[6] Ahrens ML, Maass G, Schuster P, Winkler H (1970) Kinetic study of the hydration mechanism of vitamin B6 and related compounds. J Am Chem Soc 92: 6134–6139. 5505617 10.1021/ja00724a006

[7] Peterson EA, Sober HA (1954) Preparation of crystalline phosphorylated derivatives of vitamin B6. J Am Chem Soc 76: 169–175.

[8] White RS, Dempsey WB (1970) Purification and properties of vitamin B6 kinase from Escherichia coli B. Biochemistry. 9: 4057–4064. 10.1021/bi00823a005 4917899

[9] Fu TF, di Salvo M, Schirch V (2001) Distribution of B6 vitamers in Escherichia coli as determined by enzymatic assay. Anal Biochem 298: 314–321. 11700988 10.1006/abio.2001.5401

[10] Zhao G, Winkler ME (1995) Kinetic limitation and cellular amount of pyridoxine (pyridoxamine) 5′-phosphate oxidase of Escherichia coli K-12. J Bacteriol 177: 883–891. 7860596 10.1128/jb.177.4.883-891.1995 PMC176679

[11] Safo MK, Musayev FN, di Salvo ML, Hunt S, Claude JB, et al (2006) Crystal structure of pyridoxal kinase from the Escherichia coli pdxK gene: Implications for the classification of pyridoxal kinases. J Bacteriol 188: 4542–4552. 16740960 10.1128/JB.00122-06 PMC1482971

[12] Musayev FN, Di Salvo ML, Ko TP, Schirch V, Safo MK (2003) Structure and properties of recombinant human pyridoxine 5′-phosphate oxidase. Protein Sci 12: 1455–1463. 12824491 10.1110/ps.0356203 PMC2323923

[13] Safo MK, Musayev FN, Hunt S, di Salvo ML, Scarsdale N, et al (2004) Crystal structure of the PdxY protein from Escherichia coli. J Bacteriol 186: 8074–8082. 15547280 10.1128/JB.186.23.8074-8082.2004 PMC529075

[14] Safo MK, Musayev FN, di Salvo ML, Schirch V (2001) X-ray structure of Escherichia coli pyridoxine 5′-phosphate oxidase complexed with pyridoxal 5′-phosphate at 2.0 A resolution. J Mol Biol 310: 817–826. 11453690 10.1006/jmbi.2001.4734

[15] Choi SY, Churchich JE, Zaiden E, Kwok F (1987) Brain pyridoxine-5-phosphate oxidase. modulation of its catalytic activity by reaction with pyridoxal 5-phosphate and analogs. J Biol Chem 262: 12013–12017. 3114257

[16] Li MH, Kwok F, Chang WR, Lau CK, Zhang JP, et al (2002) Crystal structure of brain pyridoxal kinase, a novel member of the ribokinase superfamily. J Biol Chem 277: 46385–46390. 12235162 10.1074/jbc.M208600200

[17] Cao P, Gong Y, Tang L, Leung YC, Jiang T (2006) Crystal structure of human pyridoxal kinase. J Struct Biol 154: 327–332. 16600635 10.1016/j.jsb.2006.02.008

[18] Newman JA, Das SK, Sedelnikova SE, Rice DW (2006) The crystal structure of an ADP complex of bacillus subtilis pyridoxal kinase provides evidence for the parallel emergence of enzyme activity during evolution. J Mol Biol 363: 520–530. 16978644 10.1016/j.jmb.2006.08.013

[19] Musayev FN, di Salvo ML, Ko TP, Gandhi AK, Goswami A, et al (2007) Crystal structure of human pyridoxal kinase: Structural basis of M(+) and M(2+) activation. Protein Sci 16: 2184–2194. 17766369 10.1110/ps.073022107 PMC2204131

[20] di Salvo ML, Hunt S, Schirch V (2004) Expression, purification, and kinetic constants for human and Escherichia coli pyridoxal kinases. Protein Expr Purif 36: 300–306. 15249053 10.1016/j.pep.2004.04.021

[21] Yang Y, Tsui HC, Man TK, Winkler ME (1998) Identification and function of the pdxY gene, which encodes a novel pyridoxal kinase involved in the salvage pathway of pyridoxal 5′-phosphate biosynthesis in Escherichia coli K-12. J Bacteriol 180: 1814–1821. 9537380 10.1128/jb.180.7.1814-1821.1998 PMC107095

[22] Yang Y, Zhao G, Winkler ME (1996) Identification of the pdxK gene that encodes pyridoxine (vitamin B6) kinase in Escherichia coli K-12. FEMS Microbiol Lett 141: 89–95. 8764513 10.1111/j.1574-6968.1996.tb08368.x

[23] Metzler CM, Metzler DE (1987) Quantitative description of absorption spectra of a pyridoxal phosphate-dependent enzyme using lognormal distribution curves. Anal Biochem 166: 313–327. 3434776 10.1016/0003-2697(87)90580-x

[24] Mehta PK, Christen P (2000) The molecular evolution of pyridoxal-5′-phosphate-dependent enzymes. Adv Enzymol Relat Areas Mol Biol 74: 129–184. 10800595 10.1002/9780470123201.ch4

[25] Christen P, Metzler DE (1985) Transaminases. John Wiley and Sons, New York, 307–362.

[26] Fonda ML (1992) Purification and characterization of vitamin B6-phosphate phosphatase from human erythrocytes. J Biol Chem 267: 15978–15983. 1322411

[27] Yang ES, Schirch V (2000) Tight binding of pyridoxal 5′-phosphate to recombinant Escherichia coli pyridoxine 5′-phosphate oxidase. Arch Biochem Biophys 377: 109–114. 10775448 10.1006/abbi.2000.1737

[28] Moccand C, Kaufmann M, Fitzpatrick TB (2011) It takes two to tango: Defining an essential second active site in pyridoxal 5′-phosphate synthase. PLoS One 6: e16042. 21283685 10.1371/journal.pone.0016042 PMC3024981

[29] Safo MK, Mathews I, Musayev FN, di Salvo ML, Thiel DJ, et al (2000) X-ray structure of Escherichia coli pyridoxine 5′-phosphate oxidase complexed with FMN at 1.8 A resolution. Structure 8: 751–762. 10903950 10.1016/s0969-2126(00)00162-3

[30] Fitzpatrick TB, Moccand C, Roux C (2010) Vitamin B6 biosynthesis: Charting the mechanistic landscape. Chembiochem 11: 1185–1193. 20397182 10.1002/cbic.201000084

[31] di Salvo ML, Safo MK, Musayev FN, Bossa F, Schirch V (2003) Structure and mechanism of Escherichia coli pyridoxine 5′-phosphate oxidase. Biochim Biophys Acta 1647: 76–82. 12686112 10.1016/s1570-9639(03)00060-8

[32] Frieden C, Kurz LC, Gilbert HR (1980) Adenosine deaminase and adenylate deaminase: Comparative kinetic studies with transition state and ground state analogue inhibitors. Biochemistry 19: 5303–5309. 7448172 10.1021/bi00564a024

[33] Morrison JF, Walsh CT (1988) The behavior and significance of slow-binding enzyme inhibitors. Adv Enzymol Relat Areas Mol Biol 61: 201–301. 3281418 10.1002/9780470123072.ch5

[34] Chattopadhyay A, Meier M, Ivaninskii S, Burkhard P, Speroni F, et al (2007) Structure, mechanism, and conformational dynamics of O-acetylserine sulfhydrylase from salmonella typhimurium: Comparison of A and B isozymes. Biochemistry 46: 8315–8330. 17583914 10.1021/bi602603c

[35] Boe AS, Bredholt G, Knappskog PM, Storstein A, Vedeler CA, et al (2004) Pyridoxal phosphatase is a novel cancer autoantigen in the central nervous system. Br J Cancer 91: 1508–1514. 15452547 10.1038/sj.bjc.6602142 PMC2409937

